# Anticipation of cyclical resource availability in the red-footed tortoise (*Chelonoidis carbonaria*): Implications for seed dispersal

**DOI:** 10.3758/s13420-025-00689-w

**Published:** 2025-09-26

**Authors:** Francesca Soldati, Oliver H. P. Burman, Elizabeth A. John, Thomas W. Pike, Anna Wilkinson

**Affiliations:** 1 Department of Life Sciences, University of Lincoln, Lincoln, LN6 7DL UK; 2 Directorate of Digital and Information Services, University of Aberdeen, Aberdeen, AB24 3FX UK

**Keywords:** Anticipation, Timing, Foraging decisions, Seed dispersal, Tortoise, Reptile

## Abstract

Many plant populations are dependent on animal-mediated seed dispersal; however, in the study of mutualistic processes, animals are generally thought of as unselective consumers. This approach is problematic as it does not consider the decisions of the foraging animals, and little attention is given to the cognitive processes that underpin these behaviors. One such process is the ability to predict fruit availability, as this would allow animals to direct their foraging towards productive food sources and would ensure rapid seed removal when fruits are ready to be eaten. This is particularly important for species that cannot move rapidly between resources, such as tortoises. This study investigated temporal (24-h cycle) and visual cues use during food anticipation in captive red-footed tortoise (*Chelonoidis carbonaria*), an important seed disperser in its natural environment. A significant increase in activity was observed in the hour immediately preceding food delivery, suggesting that the tortoises learned to predict the availability of food. Test trials, in which the visual and temporal cues were put in conflict or removed, revealed that the tortoises used both sources of information to predict food availability. Moreover, extinction trials, in which the tortoises did not have reliable temporal or visual cues prior to feeding, resulted in a rapid loss of anticipatory behavior. These findings provide insights into the cognitive processes that control anticipatory foraging behavior and, as such, have important implications for both animal and plant fitness, furthering our knowledge of mutualistic services such as seed dispersal.

**Supplementary Information:**

The online version contains supplementary material available at 10.3758/s13420-025-00689-w.

## Introduction

Animal-mediated seed dispersal plays a crucial role in plant population dynamics (Schupp et al., 2010). Surprisingly, the animal’s role in this key ecosystem process is traditionally considered to be that of a random consumer of seeds and vehicle of movement (e.g., Fragoso et al., 2003; González-Castro et al., 2019; Schupp et al., 2010). However, what an animal eats and where it chooses to go is far from random; its choices are based on information acquired through previous experience. Thus, learning and memory are likely to have a substantial and underappreciated impact upon an animal’s foraging and movement decisions and, therefore, its seed dispersal efficiency (Robira, 2024; Schupp et al., 2010).

Remembering the location of valued resources is highly adaptive as it allows an animal to relocate important items in their environment. However, because food availability can vary on a seasonal (Hamann, 2004; Momose, 2004; Moskovits & Bjorndal, 1990) or even a daily cycle (e.g., fruit falling to the ground or being replenished; Colin et al., 1992), remembering only the spatial location of a food source may not be enough to ensure access to the resource. Instead, being able to predict when fruit is likely to be available would allow an animal to direct its foraging towards food sources that are currently productive, reducing the costs associated with visiting non-productive sites (Ng et al., 2020; Wilkie et al., 1996).

Much evidence exists to suggest that animals can anticipate food availability on a daily cycle (see reviews by Crystal, 2009; Roberts, 2002; Vasconcelos et al., 2017), can learn the spatial locations of food sources (e.g., Mueller et al., 2011), and can learn visual (e.g., Schultz et al., 1997) and social (de Almeida Moura & Luchiari, 2016) cues that predict the arrival of food. For example, nectarivorous hummingbirds revisit flowers at intervals that match nectar production, indicating their ability to learn about and synchronize their foraging with these temporal patterns (Henderson et al., 2006). This strategy helps conserve energy by avoiding visits to areas devoid of nectar. Similarly, chimpanzees appear to anticipate fruit availability and may even alter their behavior on the basis of the ephemerality of the source (Janmaat et al., 2014). However, the mechanisms underlying this behavior are hard to examine in the wild (Janson & Byrne, 2007; Ng et al., 2020) as multimodal cues are associated with a plant coming into fruit, and are hard to differentiate under natural conditions (Janmaat et al., 2012). It therefore remains unknown which cues animals use when making these crucial foraging decisions or the cognitive mechanisms underpinning them.

Whereas  anticipatory behavior has been widely explored in the animal kingdom, there is very little known about this in reptiles. There is evidence of circadian rhythms in a number of species (for a review, see Tosini et al., 2001), but very little research has assessed how this translates into learned behavior. Recent work investigating the anticipation of predation risk in the grass lizard (*Takydromus viridipunctatus*) revealed that they anticipate and adapt to risk by moving from their grass night-time perches to sites that are more likely to allow avoidance of avian predators at twilight (Chen et al., 2021), suggesting that reptiles are able to adapt their behavior in anticipation of a threat. However, little is known about the mechanisms underlying this, the cues that reptiles use to do this, or the wider implications of this ability. To bridge the knowledge gap, this study considered the anticipatory behavior of tortoises, which we interpret in the light of its potential implications for understanding seed dispersal processes. This approach was inspired by Suzanne MacDonald’s work using cognition to help solve real-world challenges, from understanding raccoon problem-solving skills to design trash cans that they cannot enter, to using animal perception and preferences to adapt migratory pathways and reduce human-animal conflict (for a review, see MacDonald & Ritvo, 2016).

The red-footed tortoise (*Chelonoidis carbonaria*) is considered an important seed disperser in its natural environment (Strong & Fragoso, 2006). This species has good spatial cognition (Mueller-Paul et al., 2012; Wilkinson et al., 2007; Wilkinson et al., 2009), exhibits rapid learning (e.g., Mueller-Paul et al., 2014), can discriminate between stimuli representing food quality and quantity, and can retain this information for at least 18 months (Soldati et al., 2017) – longer than the seasonal fruiting cycle in their natural environment (Moskovits & Bjorndal, 1990). We therefore investigated whether captive red-footed tortoises were able to predict when a food source was available and, if so, the information that they used to do this. Further, as cyclic resources inevitably end, we investigated how fast this learned behavior extinguished.

To do this, each tortoise was trained to associate a specific visual cue with food prior to the onset of the experiment. During the experiment, each tortoise also had an individual feeding time, with a gap of at least 15 min between animals. At an individual’s specific feeding time their visual stimulus was introduced in the arena, and if the tortoise approached the stimulus, it was fed. After 65 days of exposure, the tortoises then received a series of tests which assessed whether they had learned about both the visual and the temporal cue, whether they could use the temporal cue in the absence of visual cues, and to test extinction of the learned response.

## Methods

### Subjects

Eleven red-footed tortoises (plastron length range: 16.7–24 cm) took part in this experiment. Some of the tortoises were captive born, and so their origin was known; however, some were rescues and so are of unknown provenance. Tortoises were housed together in a large, enriched enclosure (4 m × 3 m) and maintained on a 12-h light/dark cycle (7 a.m. to 7 p.m.). The enclosure floor was covered with soil and bark, which was dampened once a day, and the animals were provided with water ad libitum, shelters, and hot spots with both heat and UV lamps. The temperature of the room was kept between 27 ºC and 30 ºC, with humidity level ranging between 60% and 70%. During the experiment, the tortoises were fed once per day with a mixture of fresh fruit and vegetables (~150 g each).

### Materials

The visual stimuli consisted of 11 printed, laminated rectangles of paper measuring 21.0 × 29.7 cm. Each rectangle was a different color, and each tortoise was assigned a unique color cue (see Table 1). The stimuli were attached to one side of the enclosure using adhesive tags during pre-training, training, and the cue-conflict test.

**Table 1:** The order of presentation for each individual, including the time at which their food was available, the color of the visual stimulus, and the location of the food choice area. Lights were turned on at 7 a.m. and off at 7 p.m., and the camera recorded activity within the experimental arena between 9 a.m. and 4.30 p.m

Tortoise	Food delivery time	Stimulus color	Feeding location
Moses	9.45 a.m.	Yellow	Right
Wilhelmina	10.00 a.m.	Blue	Left
Aldous	10.15 a.m.	Green	Right
Betty	10.30 a.m.	Purple	Left
Alexandra	10.45 a.m.	Turquoise	Right
Savina	11.00 a.m.	Orange	Left
Patty	11.15 a.m.	Pink	Right
Darwin	11.30 a.m.	Brown	Left
Mozart	11.45 a.m.	White	Right
Seisou	12.00 p.m.	Red	Left
T19	12.15 p.m.	Black	Right

### Pretraining

Prior to the start of the experiment, the tortoises were trained to associate an individually specific visual cue with a food reward. The specific color cue used was different for each tortoise (Table 1). Pretraining took place in an experimental arena measuring 1 m × 1 m, the floor of which was entirely covered by bark. Tortoises were habituated to the arena before pre-training began. Each tortoise was trained to discriminate one colored stimulus (their S+) from the ten non-rewarding stimuli (S−), each of which represented the positive stimulus for one of the other tortoises. A two-alternative forced-choice procedure was used. In each trial, the tortoise was presented with two colored stimuli, one positive and one negative (randomly selected from the negative stimulus set). Both cues were presented together and were positioned 30 cm apart at an equal distance from the starting point (95 cm). The position of the stimuli was counterbalanced across trials.

At the onset of a trial, the tortoise was introduced to the arena in a small cage at the starting position and released after it had looked at (oriented its head towards) both stimuli; this was to ensure that it had seen both visual cues. Upon release, the tortoise had 1 min to approach one of the stimuli. The tortoises were allowed to make a single choice. A choice was counted if the tortoise approached within 5 cm of the stimulus, with its head directed towards it (Gutnick et al., 2020; Soldati et al., 2017). If the tortoise approached the correct stimulus, it was rewarded with a piece of preferred food (strawberry, mango, or dandelion), delivered with tweezers in front of the cue. If the tortoise made an incorrect choice, it was removed from the arena and placed in a holding box (30 cm × 50 cm) for 60 s. This was equivalent to the “time out” used in other studies (Miltenberger, 2012). A session consisted of 15 consecutive trials, and animals received one session per day. All tortoises had previously participated in other experiments and were therefore already trained to approach a cue to obtain a food reward. However, the color designated as the positive stimulus (S+) for each individual had not been used in prior experiments.

During this pre-training period, the tortoises received their usual amount of food, scattered around their housing enclosure after the training session of the last subject had ended. Pre-training continued until each animal reached a learning criterion of 80% correct choices in three consecutive sessions. The main experiment started once all the tortoises had reached this learning criterion.

### Training

Training took place in the enclosure where the tortoises were housed: a 4 m × 3 m rectangular arena with a centrally positioned hot spot (heat and UV lamps) along one long side and floor covered with soil and bark**.** Every day the experimenter followed a regular routine: at 9 a.m. the video camera was switched on, then tortoises were each given a warm bath, which lasted approximately 1 min for each animal. The tortoises were bathed in a specific order, which was the same order that was used for the food-delivery time during the experiment (Table 1). As the tortoises were all housed and tested together, each tortoise had a different feeding time, separated by at least 15 min. Red-footed tortoises are able to respond to (Wilkinson et al., 2010a) and use (Wilkinson et al., 2010b) social information, so this approach was used to ensure that only the experimental cues predicted food arrival and made social information less reliable. The stimuli were presented in one of two locations (either left or right) on the opposite side of the arena from the heat lamps. This was consistent for an individual but differed across tortoises. Each location had a choice area which consisted of a black rubber mat (50 cm × 70 cm) with a white line marking 20 cm × 50 cm area in front of the visual cue presentation position. The area within the line was defined as the choice area, and a tortoise was considered to have made a correct choice if its head entered this area.

A trial consisted of the presentation of an individual’s color cue, at its food delivery time, in its specific feeding location. The tortoise then had 2 min to approach the choice area. If the tortoise made a correct choice, the visual cue was removed, and the tortoise was picked up and placed in a feeding area outside the enclosure. This was to ensure that no other tortoises had access to any reward. The tortoises received their daily feed of mixed fresh fruit, vegetables, and leaves whilst in the feeding area. The tortoise remained in the feeding area until the food delivery time of the next individual.

If the tortoise did not approach the choice area at its food delivery time then, after the 2-min trial time was up, the individual was placed 1 m from the stimulus. The tortoise then had 1 min to approach the choice area. If it entered the choice area then the tortoise was removed from the enclosure and fed as described above. If it still did not approach the choice area, the cue was removed and the tortoise remained in the enclosure. If the tortoise did not approach the stimulus on consecutive days, it was then removed from the enclosure at its feeding time, the cue was transferred into the feeding area, and the tortoise was fed in proximity to it. This ensured that all the individuals maintained a normal body weight and represents a standard husbandry regime for this species (e.g., Pingleton 2001).

The enclosure (and activity within it) was filmed from 9 a.m. until 4.30 p.m. using a wide-angled camera (GoPro HERO3+, 720p resolution, 60 fps; see Online Supplementary Material). The 24-h feeding cycle continued for 65 days.

### Testing

Upon completion of the training, tortoises were presented with three different tests.

#### Cue-conflict test

This test investigated whether the tortoises had learned about temporal cues (feeding time) or visual cues (colored stimuli), or both. To assess this, an individual’s visual stimulus was presented at an incorrect time. The procedure was the same as described in training, except that the tortoises were not removed from the arena and did not receive any reward. This test was repeated three times, each separated by 3 days of training (intermixed training days). If tortoises anticipate the food delivery time using exclusively temporal cues, the prediction was that they would not respond to the color cue when it was introduced at the unanticipated time. If tortoises exclusively used the visual cue as a signal for food delivery, we predicted that they would respond to the color cue no matter what time it was introduced in the arena and with the same latency of response as during training. If tortoises were using both temporal and visual cues to anticipate the food delivery event, it would be predicted that they would be more active around the expected food delivery time; if they did respond to the visual cue, the latency of the response would be longer than in the training, because its appearance would be unexpected.

#### Absence-of-cue test

This test further examined the impact of visual cues, and the events related to their introduction in the arena on anticipatory behavior. During this test the tortoises were bathed at the start of the day as they were on a training trial. However, no further visual or movement cues were presented. The experimenter did not approach the arena at any time after turning the camera on, tortoises were not removed, and no other interactions took place. The test was repeated three times, each separated by 3 days of training (intermixed training days). If tortoises are able to use just temporal cues to anticipate the food delivery time, then it would be predicted that they would be equally active on test days at the time approaching their feeding time as they would be on the intermixed training days.

#### Extinction test

As food availability varies and resources from fruiting trees are transient, we wanted to assess how rapidly the tortoises learned that their cyclical feeding time was no longer reliable. This test, therefore, examined whether the tortoises rapidly extinguished their anticipatory behavior after the removal of both temporal and visual cues. This test was run for 6 days, and the tortoises did not receive any of the cues that they could have used in the training: they did not receive a morning bath, visual cues were not presented, and they were fed together at least 1 h after the last learned reward time. It was predicted that if the tortoises were able to rapidly extinguish their learning, there would be a reduction in their anticipatory behavior between the first 3 extinction days and the final 3 extinction days.

### Data analysis

Three subjects were excluded from the data analysis because, although they successfully reached the learning criterion during the pre-training phase, they never completed the task without experimenter intervention during training. Thus, data from the remaining eight tortoises were analyzed.

To investigate whether tortoises learned to anticipate their food delivery time, the activity of the tortoises during the initial 3 and final 3 days of the 65 days of training was compared. Specifically, we coded tortoises during the 75 min prior to their specific food delivery time as either “active” (i.e., walking) or “inactive” for each minute; that is consistent with previous studies on mice showing that food anticipatory activity is expressed approximately within the hour before the food delivery time (e.g., Luby et al., 2012; Martini et al., 2018). Time prior to food delivery was grouped into five periods of equal duration for the analysis: 74–60, 59–45, 44–30, 29–15, and 14–0 min before delivery. We then fitted a generalized linear mixed-effects model (GLMM), using the *glmer* function in *lme4* package (Bates et al., 2015) for R v.4.4.0 (R Core Team, 2024), with a binary dependent variable of active/inactive, training period (the initial 3 days and final 3 days of training) and time period before food delivery (and their interaction) as predictors, and study day and subject identity as random effect terms. Significance of the fixed effects was assessed using the *Anova* function in the *car* package (Fox & Weisberg, 2019), and post hoc tests were performed with the *emmeans* package (Lenth et al., 2025). Where multiple testing occurred, p-values were adjusted using Bonferroni correction (Dunn, 1961).

The cue-conflict test, the absence-of-cue test, and the extinction test were all analyzed in an almost identical way to the training trials, the only difference being that comparisons were made between the 3 test days and the 3 intermixed training days preceding each test day (for the cue-conflict and absence-of-cue tests) and the initial and final 3 days of the test (for the extinction test). In addition, for the cue conflict test, we analyzed the extent to which tortoises approached their correct color cue (presented at the incorrect time) as a function of phase (test or intermixed training) using a GLMM with a binary response (approached/did not approach) and study day and subject identity as random effects. However, because this model failed to converge when fitted with the *glmer* function, this analysis was instead run using the *bglmer* function in the *blme* package (Chung et al., 2013) with an uninformative normal prior for the fixed effect. We also considered the tortoises’ latency to respond to their correct color cue (in s, from the introduction of the cue to the moment in which the respective tortoise started to walk towards it) during test compared to intermixed training using a linear mixed-effects model (LMM), in which study day and subject identity were included as random-effect terms. Tortoises that never approached the cue were excluded from this analysis, and latency was normalized using a Johnson transformation (Chou et al., 1998) to ensure that the model assumptions of normally distributed and homoscedastic residuals were met.

## Results

**Fig. 1:**
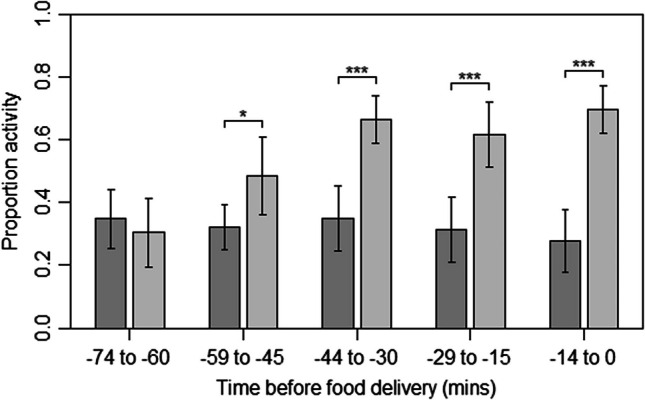
Mean ± SE proportion of activity observed in the 75 min prior to each tortoise’s specific food delivery time, in 15-min periods, between the initial 3 days (dark gray) and final 3 days (light gray) of training. Significant pairwise differences between initial and final training days within a time period are denoted by asterisks: * *p* < 0.05; *** *p* < 0.001

The activity of the tortoises during training was significantly predicted by the interaction between training period (the initial 3 days and final 3 days of training) and time before food delivery (χ^2^(4) = 84.79,* p* < 0.001). Specifically, while there was no temporal change in activity during the initial 3 days of training (linear contrast over successive time periods prior to food delivery: *z* = −2.43, *p* = 0.061), by the final 3 days of training tortoises showed a significant increase in activity as the food delivery time approached (linear contrast: *z* = 9.69, *p* < 0.001) such that activity levels were significantly higher in the four time periods immediately prior to food delivery (all *ps* < 0.05) (Fig. 1).

### Cue-conflict test

**Fig. 2:**
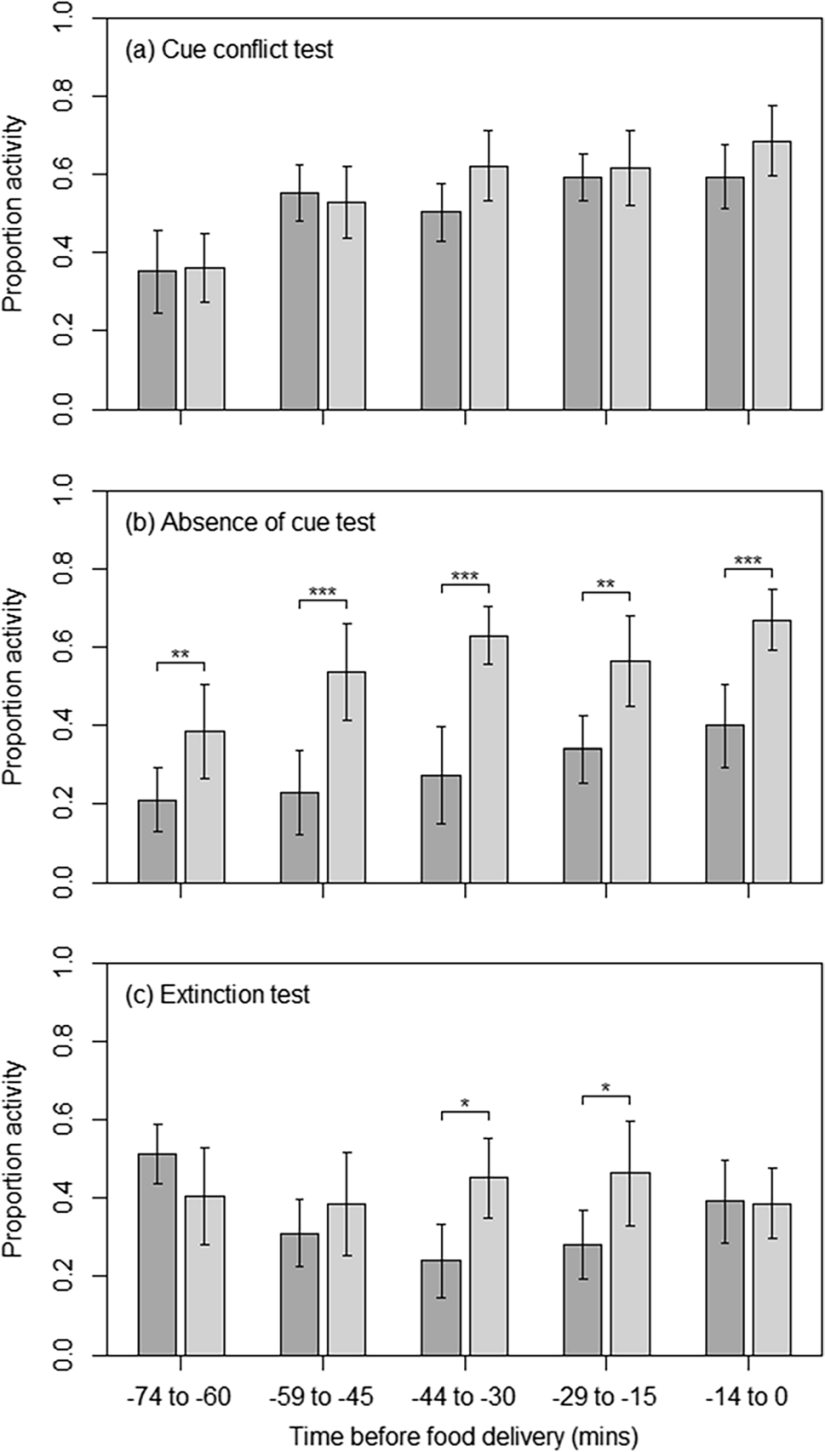
The mean ± SE proportion of activity observed in the 75 min prior to each tortoise’s specific food delivery time, in 15-min blocks, for (**a**) the cue-conflict test trials (dark gray) and the intermixed training trials (light gray), (**b**) the absence-of-cue test trials (dark gray) and the intermixed training trials (light gray), and (**c**) the initial 3 (dark gray) and final 3 days (light gray) after the food delivery time changed in the extinction test. Significant pairwise differences between conditions within a time block are denoted by asterisks: * *p* < 0.05; ** *p* < 0.01; *** *p* < 0.001

**Fig. 3:**
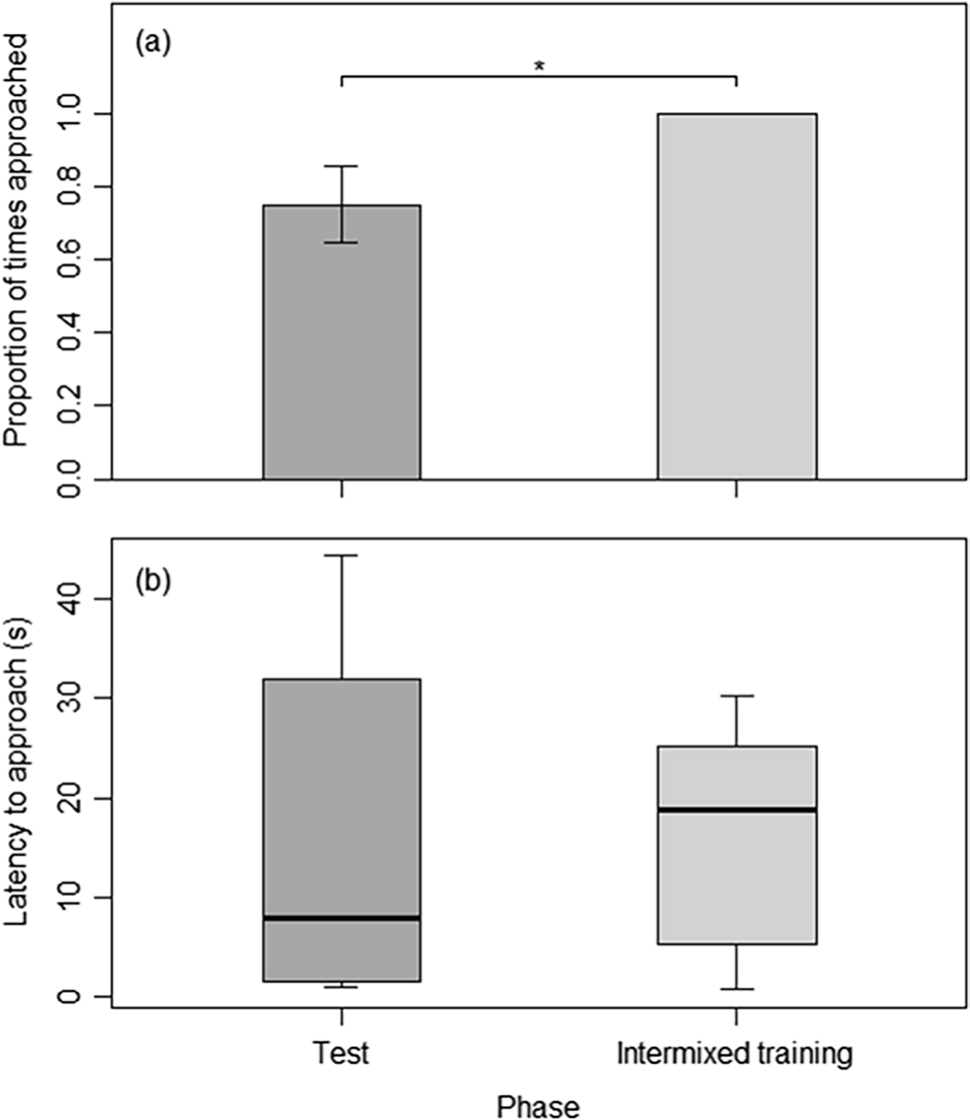
(**a**) The mean ± SE proportion of time tortoises approached the correct cue during the cue conflict test (dark gray) compared to the intermixed training trials (light gray). (**b**) The latency to response to the visual cue during the cue conflict test (dark gray) compared to during the intermixed training trials (light gray). Significant pairwise differences between phases are denoted by asterisks: * *p* < 0.05

During the cue-conflict test, the activity of the tortoises was not predicted by the interaction between phase (test or intermixed training) and the time leading up to food delivery (χ^2^(4) = 9.08,* p* = 0.059) or by the main effect of phase (χ^2^(1) = 1.27,* p* = 0.260); however, during both test and intermixed training trials, there was a significant increase in activity as the anticipated food delivery time approached (χ^2^(4) = 78.74,* p* < 0.001) (Fig. 2a). Tortoises visited their correct visual cue significantly less often during cue-conflict test trials than during intermixed training trials (χ^2^(1) = 4.39, *p* = 0.036; Fig. 3a), but when they did, the latency to respond did not differ (χ^2^(1) = 0.02, *p* = 0.876; Fig. 3b).

### Absence-of-cue test

During the absence-of-cue test, activity was significantly predicted by the interaction between phase (test or intermixed training) and the time prior to food delivery (χ^2^(4) = 10.38,* p* = 0.034). Whereas there was a significant increase in activity as the food delivery time approached during both test (linear contrast: *z* = 9.69, *p* < 0.001) and intermixed training trials (linear contrast: *z* = 9.69, *p* < 0.001), activity was consistently and significantly higher during intermixed training for all time periods (all *ps* < 0.05) (Fig. 2b).

### Extinction test

The activity of the tortoises in the initial 3 and final 3 days of the extinction test was significantly predicted by the interaction between phases (initial and final 3 days of the test) and time before food delivery (χ^2^(4) = 56.89,* p* < 0.001). Specifically, while tortoises showed a significant curvilinear increase in activity as the food delivery time approached during the initial 3 days of the test (quadratic contrast: *z* = 7.51, *p* < 0.001), albeit much reduced compared to during training, by the final 3 days there was no temporal change in activity levels (linear contrast: *z* = 0.23, *p* = 0.998) (Fig. 2c), suggesting that the anticipatory activity of tortoises extinguished in the 3 days after the food delivery time was changed.

## Discussion

Our results reveal that the tortoises used both temporal and visual information to anticipate food delivery, with their activity levels increasing substantially in the hour before food delivery. The cue-conflict test showed that activity patterns followed a similar pattern to training, but that there was a significant decrease in response to the visual cues, suggesting that the tortoises were using temporal information. Whilst the tortoises did go to the visual cues significantly less during the cue-conflict test than in training, when the tortoises observed the cues, their latency to respond was the same as was seen during training, suggesting that the tortoises also used visual information. Further, when the visual cues were entirely absent, the tortoises still showed temporal anticipation; however, overall activity was significantly decreased. It appears that the temporal cue resulted in greater arousal in the period prior to the food availability, and the visual cue led to approach behavior, suggesting that animals can use complementary cues when making foraging decisions.

These results therefore have important ecological implications as this sort of anticipatory behavior would enhance foraging efficiency by conserving energy expenditure until the time when the food availability is approaching (Henderson et al., 2006). Such anticipation could potentially allow the animal to reach the food before competitors, something vital for a relatively slow animal that lives in an ecologically complex and competitive environment (Morales et al., 2013; Moskovits & Bjorndal 1990; Ng et al., 2020). Successful prediction of resource availability is also likely to impact upon plant fitness, leading to an increase in the seed removal rate of plants by seed dispersers, and a consequent decrease of seed predation, and increasing the likelihood of seeds being removed from under the parent plant (González-Varo et al., 2018; John et al., 2016).

As the fruiting period of a plant is limited (Hamann, 2004; Moskovits & Bjorndal 1990), it is important that animals are able to rapidly adapt their behavior once the fruiting period is over (Moore et al., 2011). The extinction test revealed that when cues were removed for just 3 days, the tortoises readily lost their anticipatory behavior, demonstrating behavioral flexibility (Bridgerman & Tattersall, 2019). This rapid extinction of the learned behavior may also benefit the plant as the animal will be motivated to move away, reducing the likelihood of dispersal close to the parental plant.

Our findings are among the first to provide evidence of anticipatory behavior in reptiles and are comparable to that observed in mammals and birds (Mueller et al., 2011; Roberts, 2002; Wilkie et al., 1996). Further, we reveal that the tortoises use both temporal and visual information to anticipate resource availability. Understanding the cues involved in anticipatory behavior can allow us to better understand the role that cognition plays on key ecosystem processes such as seed dispersal (John et al., 2016; Robira, 2024).

## Supplementary Information

Below is the link to the electronic supplementary material.

Supplementary file1 (XLSX 276 KB)

## Data Availability

The data are available as electronic supplementary information.

## Code availability

Analysis code may be shared upon reasonable request to the corresponding author.
